# The cytokine polymorphisms affecting Th1/Th2 increase the susceptibility to, and severity of, chronic ITP

**DOI:** 10.1186/s12865-017-0210-3

**Published:** 2017-05-16

**Authors:** Noriyuki Takahashi, Takayuki Saitoh, Nanami Gotoh, Yasuhiro Nitta, Lobna Alkebsi, Tetsuhiro Kasamatsu, Yusuke Minato, Akihiko Yokohama, Norifumi Tsukamoto, Hiroshi Handa, Hirokazu Murakami

**Affiliations:** 1 Graduate School of Health Sciences, Gunma University, 3-39-22 Showa-machi, Maebashi, Gunma 371-8514 Japan; 2 Department of Virology and Preventive Medicine, Gunma University, Gunma, Japan; 3 Division of Blood Transfusion Service, Gunma University Hospital, Gunma, Japan; 4 Oncology Center, Gunma University Hospital, Gunma, Japan; 5 Department of Medicine and Clinical Science, Gunma University Graduate School of Medicine, Gunma, Japan

**Keywords:** Immune thrombocytopenic purpura, Polymorphism, Th1 cells, Cytokine, Cytokine receptor

## Abstract

**Background:**

T-helper cell type 1 (Th1) polarization in chronic immune thrombocytopenia (cITP) has been reported at the protein and mRNA levels. We evaluated the impact of Th1/Th2 cytokine and cytokine receptor functional polymorphisms on both susceptibility to, and severity of, cITP. We analysed *IFN-γ* + 874 T/A, *IFN-γR* -611G/A, *IL-4* -590C/T, and *IL-4Rα* Q576R polymorphisms in 126 cITP patients (male/female: 34/92; median age: 47.7 years) and 202 healthy control donors. Genotyping was determined by PCR and direct sequencing. The Th1/Th2 ratio was detected in peripheral blood mononuclear cells via flow cytometry.

**Results:**

cITP patients had a higher frequency of the *IL-4Rα* 576 non-QQ genotype compared to healthy subjects (*P* = 0.04). cITP patients with the *IFN-γ* +874 non-AA genotype (high expression type) showed more severe thrombocytopenia than those with the AA genotype (*P <* 0.05). cITP patients had a significantly higher Th1/Th2 ratio than control patients (*P* < 0.01); this ratio was inversely correlated with platelet counts. Furthermore, patients with both *IFN-γ* +874 non-AA genotype (high expression type) and *IFN-γR −*611 non-AA genotype (high-function type) had a significantly higher Th1/Th2 ratio (*P* < 0.05).

**Conclusions:**

The cytokine polymorphisms affecting Th1/Th2 increase the susceptibility to, and severity of, chronic ITP.

## Background

Chronic immune thrombocytopenia (cITP) is an acquired immune-mediated disorder characterized by isolated thrombocytopenia. Several immune mechanisms contribute to the pathogenesis of cITP, including increased platelet destruction in the reticuloendothelial system and impaired platelet production in the bone marrow [1]. Recent studies have revealed that T helper (Th) type 1 (Th1) cytokine polarization occurs in cITP patients [2–5]; several investigators have also reported that the Th1/Th2 ratio is inversely correlated with disease progression [3].

Naive CD4+ T cells differentiate into several different Th cells, including Th1, Th2, Th17, and regulatory T (Treg) cells. Th cell functions are determined by their cytokine secretion patterns; Th1 and Th2 cells are defined by their ability to produce interferon-γ (IFN-γ) but not interleukin (IL)-4, and IL-4 but not IFN-γ, respectively [6–8]. Many investigators have shown IFN-γ to be upregulated at both the mRNA and protein levels in cITP patients [3, 9]. It was also reported that serum levels of IL-4 were decreased in cITP patients [9, 10]. Such findings demonstrate that Th1 polarization is consistent with characteristics of cITP. However, it remains unclear whether the influences of Th1/Th2 cytokines on cITP are due to genetic factors.

We investigated the existence of a role for Th1/Th2 cytokine and cytokine receptor functional polymorphisms, including *IFN-γ* +874 T/A, *IFN-γR* -611G/A, *IL-4* -590C/T, and *IL-4Rα* Q576R, on susceptibility to cITP, as well as on its clinical features. Furthermore, we explored the association between the Th1/Th2 ratio and these polymorphisms in both healthy donors and cITP patients.

## Methods

### Patients and control subjects

In this study, 126 Japanese cITP patients (92 females and 34 males with a median age of 47.7 [range: 2.4–82.3] years), as well as 202 race- and sex-matched healthy subjects were examined. cITP was defined as isolated thrombocytopenia (platelet count <100 × 10^9^/L) in the absence of other causes or disorders that may be associated with thrombocytopenia according to the criteria of the ITP International Working Group (IWG) [11]. cITP was also diagnosed if the disease lasted longer than 12 months [11]. “Very severe thrombocytopenia” was defined as a platelet count <10 × 10^9^/L at presentation of cITP. “Severe bleeding tendency” was defined as gastrointestinal bleeding and cerebral haemorrhage [11]. The responses were assessed according to the criteria of the ITP IWG [11]. In these guidelines, a complete response was defined as a platelet count of at least 100 × 10^9^/L, and a response was defined as a platelet count between 30 and 100 × 10^9^/L on condition that it was at least double the baseline count. “Loss of R” was defined as a platelet count <30 × 10^9^/L, a less than 2-fold increase in platelet count from baseline, or the presence of bleeding. “Corticosteroid-dependence” was defined as the ongoing need for continuous corticosteroid administration or frequent courses of corticosteroids to maintain a platelet count at or above 30 × 10^9^/L and/or to avoid bleeding [11]. “Severe cITP” was defined by the presence of bleeding symptoms at presentation of severity sufficient to mandate treatment, or by the occurrence of new bleeding symptoms requiring additional therapeutic intervention via increasing the dose of the platelet-enhancing agent or replacing the agent [11]. “Refractory ITP” was defined as failure to achieve at least R or loss of R after splenectomy [11]. “Second-line treatment” was defined as additional therapy beyond glucocorticoids.

### Genotyping

The *IL-4* -590C/T (rs2243250), *IL-4Rα* Q576R (rs1801275), and *IFN-γR* -611G/A (rs1327474) genotypes were determined using the polymerase chain reaction restriction fragment length polymorphism method. Genomic DNA was extracted from whole blood using a DNA Isolation kit (Qiagen GmbH, Hilden, Germany). The reaction volume was 20 μL, comprising 1 μL of genomic DNA, 0.2 mM of dNTPs, 0.5 U of TaKaRa Ex Taq HS DNA polymerase (TaKaRa Bio, Japan), and 0.5 μM of each of 2 primers. We used the primers 5'- ACTAGGCCTCACCTGATACG -3' (forward) and 5'- GTTGTAATGCAGTCCTCCTG -3' (reverse) [12] for analysis of IL-4 -590C/T, the primers 5'- GCCCCCACCAGTGGCTACC -3' (forward) and 5'- GAGGTCTTGGAAAGGCTTATAC -3' (reverse) [13] for analysis of IL-4Rα Q576R, the primers 5'- CTCTTCATGAGAGGCTGTCT -3' (forward) and 5'- TAACTCTTGGAGTTCACCTGG -3' (reverse) [14] for analysis of IFN-γR -611G/A. Identification of the alleles at each polymorphic site was performed by incubating the PCR product with the restriction enzyme BsmFI (*IL-4*), MspI (*IL-4Rα*), and Hpy188I (*IFN-γR*) (New England Biolabs, Ipswich, MA, USA) followed by electrophoresis through a 2.0% agarose gel (for *IL-4*) or a 6% polyacrylamide gel (for *IL-4Rα* and *IFN-γR*).

### Genotyping by allele-specific PCR

The *IFN-γ* +874 T/A (rs2430561) genotype was determined using the allele-specific PCR method. Genomic DNA was extracted from whole blood using a DNA Isolation kit (Qiagen). The reaction volume was 20 μL, comprising 1 μL of genomic DNA, 0.2 mM of dNTPs, 0.5 U of TaKaRa Ex Taq HS DNA polymerase (TaKaRa Bio, Japan), and 0.5 μM of each of 3 primers: 5'-TCA ACA AAG CTG ATA CTC CA-3' (common reverse), 5'-TTC TTA CAA CAC AAA ATC AAA TCT -3' (T allele specific forward), and 5'-TTC TTA CAA CAC AAA ATC AAA TCA-3' (A allele specific forward) [15]. The amplified product was analysed by electrophoresis on a 2% agarose gel.

### Genotyping by sequencing

To confirm the accuracy of our assays, several PCR products were sequenced and analysed using an ABI Prism Genetic Analyzer (Applied Biosystems, CA, USA).

### Flow cytometry for analysis of the Th1/Th2 ratio

We determined the Th1/Th2 ratio using flow cytometry as previously described by Ogawawa et al. [2]*.* Whole heparinized blood was added to an equal volume of RPMI 1640 medium (Gibco, Grand Island, NY, USA) in 15 ml centrifuge tubes. Twenty-five ng/mL of phorbol 12-myristate 13-acetate and 1 μg/mL of ionomycin (Sigma-Aldrich, St. Louis, MO, USA) were added to all tubes except the resting controls; all tubes were supplemented with 10 μg/mL Brefeldin A (Sigma-Aldrich) except the activation controls. Tubes were incubated at 37 °C in 7% CO_2_ for four hours. After incubation with FACS lysing solution and FACS permeabilizing solution, cells were stained at 4 °C for 30 min with antihuman CD4-PE-Cy5 (BD Biosciences, CA, USA), FastImmune™ IFN-γ FITC/IL-4 PE (BD Biosciences) for intracellular cytokine staining and CD69 PE (BD Biosciences) for activation markers. FastImmune™ IgG2a FITC/IgG1 PE isotype control (BD Biosciences) and mouse IgG1 PE control (BD Biosciences) were used as negative controls. Three-color flow cytometric analysis was performed on a FACS Canto flow cytometer (BD Biosciences) using the FACS Diva software (BD Biosciences). Cells were logically gated on CD4 vs. side-scattered light (SSC) and forward-scattered light vs. SSC. Data were analysed using the FACS Diva software and displayed as dot plots of *IFN-γ* FITC vs. *IL-4* PE. *IFN-γ* ^*+*^and *IL-4* ^*−*^ cells were defined as Th1 cells, while *IFN-γ* ^*−*^ and *IL-4* ^*+*^ cells were deemed Th2 cells. Analysis of the stimulation effect was based on the fraction of CD69-positive cells after activation, as evaluated using histograms. In all analyses, CD69 positivity exceeded 95%.

### Statistical analysis

The measured continuous data were expressed as mean ± standard deviation. Allele and genotype frequencies were analysed using the chi-square test, and clinical characteristics and treatment response were analysed using the chi-square test and student’s *T* test. The Th1/Th2 ratio and age were determined using the non-parametric Mann–Whitney *U* test. *P*-values were two-tailed, and *P*-values < 0.05 were considered statistically significant. We also compared the genotype frequencies with those calculated using the Hardy-Weinberg equilibrium theory (*p*2 + *q*2 + 2*pq* = 1, where q is the variant allele frequency).

## Results

### Patients’ characteristics

Of 126 patients, 92 were female (73.0%) and 34 were male (27.0%). Their median age at diagnosis was 47.7 years (range, 2.4–82.3 years). The platelet count ranged from 1.0 × 10^9^/L to 96.0 × 10^9^/L with a median count of 20.0 × 10^9^/L at the initial diagnosis. The patients’ characteristics are shown in Table 1.

**Table 1:** Characteristics of patients with chronic ITP

No. of patients (female/male)	126	(92/34)
Age (years) at diagnosis, range (median)	2.4–82.3	(47.7)
Platelet count (×10^9^/L) at diagnosis, range (median)	1.0–96.0	(20.0)
Mild thrombocytopenia, no. of cases (%)	28	(22.2)
Moderate thrombocytopenia, no. of cases (%)	28	(22.2)
Severe thrombocytopenia, no. of cases (%)	40	(31.7)
Very severe thrombocytopenia, no. of cases (%)	30	(23.8)
Minimum platelet count (×10^9^⁄L), mean ± SD	18.8	±18.0
Bleeding tendency, no. of cases (%)	80	(63.5)
Severe bleeding tendency, no of cases (%)	3	(2.4)
Severe ITP, no. of cases (%)	24	(19.0)
Treatment, no. of cases (%)	98	(77.8)
Prednisolone, no. of cases (%)	82	(65.1)
Splenectomy, no. of cases (%)	19	(15.1)
Eradication of *Helicobacter pylori,* no. of cases (%)	43	(34.1)

Mild thrombocytopenia: >50 × 10^9^⁄L, moderate thrombocytopenia: 30 × 10^9^–50 × 10^9^⁄L, severe thrombocytopenia: 10 × 10^9^–30 × 10^9^⁄L, very severe thrombocytopenia: <10 × 10^9^⁄L, minimum platelet count: minimum platelet count during clinical course, Severe ITP is defined by the presence of clinical significant bleeding symptoms at presentation sufficient to mandate treatment, *ITP* immune thrombocytopenia

### Genotype and allele frequencies of IFN-γ, IFN-γR, IL-4, and IL-4Rα polymorphism in patients with cITP and healthy controls

The genotype and allele frequencies of *IFN-γ* +874 T/A, *IFN-γR* -611G/A, *IL-4* -590C/T, and *IL-4Rα* Q576R are shown in Table 2. The genotype distributions of these four polymorphisms in healthy subjects were similar to those observed in previous studies of Japanese populations [16–21]. Patients with cITP had a significantly lower frequency of the *IL-4Rα* 576 QQ genotype compared to healthy controls using a dominant model (69.8% vs. 79.7% respectively, odds ratio [OR] = 0.59, 95% confidence interval [CI] = 0.35–0.98, *P* = 0.04). However, no significant differences in the genotype frequencies of *IFN-γ*, *IFN-γR*, and *IL-4* were observed between cITP patients and healthy controls using a dominant model and recessive model. Genotype frequencies of the four polymorphisms were in Hardy-Weinberg equilibrium in both cITP patients and healthy controls. These data show that *IL-4Rα* Q576R is associated with susceptibility to cITP.

**Table 2:** Genotype distribution and allele frequency of polymorphism in patients with chronic ITP and healthy controls

			ITP (%)	Control (%)	*P* value	OR	95%CI	Statistical model
IFN-γ	Genotype	TT	3 (2.4)	2 (1)	0.38	2.44	0.40–14.80	Recessive
		TA	15 (11.9)	44 (21.8)				
		AA	108 (85.7)	156 (77.2)	0.06	1.77	0.97–3.22	Dominant
	Alleles	T	21 (8.3)	48 (11.9)	0.15	0.67	0.39–1.16	
		A	231 (91.7)	356 (88.1)		1.48	0.87–2.54	
FN-γR	Genotypes	GG	0 (0)	0 (0)	-	-	-	
		GA	11 (8.7)	22 (10.9)				
		AA	115 (91.3)	180 (89.1)	0.53	1.28	0.60–2.73	Dominant
	Alleles	G	11 (4.4)	22 (5.4)	0.54	0.79	0.38–1.66	
		A	241 (95.6)	382 (94.6)		1.26	0.60–2.65	
IL-4	Genotypes	CC	17 (13.5)	17 (8.4)	0.14	1.70	0.83–3.46	Recessive
		CT	56 (44.4)	88 (43.6)				
		TT	53 (42.1)	97 (48.0)	0.29	0.79	0.50–1.23	Dominant
	Alleles	C	90 (35.7)	122 (30.2)	0.14	1.28	0.92–1.79	
		T	162 (64.3)	282 (69.8)		0.78	0.56–1.09	
IL-4Rα	Genotypes	QQ	88 (69.8)	161 (79.7)	0.04	0.59	0.35–0.98	Dominant
		QR	37 (29.4)	37 (18.3)				
		RR	1 (0.8)	4 (2.0)	0.65	0.40	0.04–3.58	Recessive
	Alleles	Q	213 (84.5)	359 (88.9)	0.11	0.69	0.43–1.09	
		R	39 (15.5)	45 (11.1)		1.46	0.92–2.32	

*ITP*: immune thrombocytopenia, *OR:* odds ratios, 95% *CI:* 95% confidence intervals, *IFN*: interferon, *IL:* interleukin, Recessive: Recessive model, Dominant: Dominant model

### The clinical characteristics of the patients with cITP and the treatment response according to IFN-γ, IFN-γR, IL-4, and IL-4Rα polymorphisms

We examined the association between the polymorphisms and the clinical characteristics of cITP patients (Tables 3 and 4). cITP patients with *IFN-γ* +874 non-AA genotype (high expression type) showed a lower minimum platelet count than those with an AA genotype (12.9 × 10^9^/L ± 10.7 × 10^9^/L vs 19.4 × 10^9^/L ± 18.9 × 10^9^/L, *P* = 0.045). However, there was no significant association between the other three genotype distributions and their various clinical features. We also explored the association between the four polymorphisms and treatment response (Tables 5 and 6). cITP patients with the *IL-4* -590 cm^3^ genotype (low expression type) had a higher incidence of second-line treatment than those with non-CC genotypes (52.9% vs 25.7% respectively, OR = 3.25, 95% CI = 1.15–9.25, *P* = 0.04). No significant difference in treatment response was observed in other genotype distributions. These data suggest that Th1 polarization due to Th1/Th2 gene polymorphisms plays a role in the clinical features of cITP was well as in the response to treatment.

**Table 3:** Clinical characteristics of chronic ITP patients with IFN-γ and IFN-γR

	IFN-γ AA		IFN-γ non-AA					IFN-γR AA		IFN-γR non-AA				
	*N*	(%)	*N*	(%)	*P* value	OR	95% CI	*N*	(%)	*N*	(%)	*P* value	OR	95% CI
No. of patients (%)	108	(85.7)	18	(14.3)				115	(91.3)	11	(8.7)			
Female patients, no. of cases (%)	82	(75.9)	10	(55.6)	0.09	2.52	0.90–7.06	84	(73.0)	8	(72.7)	1.00	1.02	0.25–4.08
Age (years) at diagnosis, range (median)	2.4–81.9	(49.0)	5.0–82.3	(37.7)	0.25			2.4–82.3	(46.0)	19.9–79.0	(60.9)	0.20		
Platelet count (×10^9^/L) at diagnosis, mean ± SD	30.8	± 24.9	21.9	± 20.7	0.15			29.9	± 24.6	25.8	± 22.8	0.60		
Mild thrombocytopenia, no. of cases (%)	26	(24.1)	2	(11.1)	0.36	2.54	0.55–11.77	26	(22.6)	2	(18.2)	1.00	1.32	0.27–6.47
Moderate thrombocytopenia, no. of cases (%)	24	(22.2)	4	(22.2)	1.00	1.00	0.30–3.32	25	(21.7)	3	(27.3)	0.71	0.74	0.18–3.00
Severe thrombocytopenia, no. of cases (%)	35	(32.4)	5	(27.8)	0.79	1.25	0.41–3.77	37	(32.2)	3	(27.3)	1.00	1.27	0.32–5.05
Very severe thrombocytopenia, no. of cases (%)	23	(21.3)	7	(38.9)	0.13	0.43	0.15–1.22	27	(23.5)	3	(27.3)	0.72	0.82	0.20–3.30
Minimum platelet count (×10^9^⁄L), mean ± SD	19.4	± 18.9	12.9	± 10.7	0.045			18.2	± 17.6	21	± 23.1	0.63		
Bleeding tendency, no. of cases (%)	69	(63.9)	11	(61.1)	0.80	1.13	0.40–3.14	73	(63.5)	7	(63.6)	1.00	0.99	0.28–3.59

**Table 4:** Clinical characteristics of chronic ITP patients with IL-4 and IL-4Rα

	IL-4 CC		IL-4 non-CC					IL-4Rα QQ		IL-4Rα non-QQ				
	*N*	(%)	*N*	(%)	*P* value	OR	95% CI	*N*	(%)	*N*	(%)	*P* value	OR	95% CI
No. of patients (%)	17	(13.5)	109	(86.5)				88	(69.8)	38	(30.2)			
Female patients, no. of cases (%)	16	(94.1)	76	(69.7)	0.04	6.95	0.88–54.57	65	(73.9)	27	(71.1)	0.74	1.15	0.49–2.69
Age (years) at diagnosis, range (median)	10.5–82.3	(49.1)	2.4–81.9	(47.4)	0.85			2.4–82.3	(46.6)	17.1–79.2	(54.9)	0.16		
Platelet count (×10^9^/L) at diagnosis, mean ± SD	24.5	± 22.4	30.3	± 24.7	0.36			29.8	± 24.8	28.9	± 23.9	0.84		
Mild thrombocytopenia, no. of cases (%)	3	(17.6)	25	(22.9)	0.76	0.72	0.19–2.71	18	(20.5)	10	(26.3)	0.47	0.72	0.30–1.75
Moderate thrombocytopenia, no. of cases (%)	3	(17.6)	25	(22.9)	0.76	0.72	0.19–2.71	22	(25.0)	6	(15.8)	0.35	1.78	0.66–4.82
Severe thrombocytopenia, no. of cases (%)	6	(35.3)	34	(31.2)	0.78	1.20	0.41–3.52	26	(29.5)	14	(36.8)	0.42	0.72	0.32–1.60
Very severe thrombocytopenia, no. of cases (%)	5	(29.4)	25	(22.9)	0.55	1.40	0.45–4.36	22	(25.0)	8	(21.1)	0.82	1.25	0.50–3.13
Minimum platelet count (×10^9^⁄L), mean ± SD	16.4	± 19.7	18.8	±17.8	0.61			17.5	± 17.4	20.8	± 19.4	0.35		
Bleeding tendency, no. of cases (%)	11	(64.7)	69	(63.3)	1.00	1.06	0.37–3.09	55	(62.5)	25	(65.8)	0.73	0.87	0.39–1.92

Mild thrombocytopenia: >50 × 109⁄L, moderate thrombocytopenia: 30 × 109–50 × 109⁄L, severe thrombocytopenia: 10 × 109–30 × 109⁄L, very severe thrombocytopenia: <10 × 109⁄L, minimum platelet count: minimum platelet count during the clinical course. *ITP* immune thrombocytopenia, *IFN* interferon, *IL* interleukin, *OR* odds ratio, *CI* confidence interval, *SD* standard deviation

**Table 5:** Treatment response of chronic ITP patients with IFN-γ and IFN-γR

	IFN-γ AA		IFN-γ non-AA					IFN-γR AA		IFN-γR non-AA				
	*N*	(%)	*N*	(%)	*P* value	OR	95% CI	*N*	(%)	*N*	(%)	*P* value	OR	95%
All treatment, no. of cases (%)	82	(75.9)	15	(83.3)	0.76	0.66	0.18–2.48	89	(77.4)	8	(72.7)	0.71	1.35	0.33–5.47
Second line treatment, no. of cases (%)	33	(30.6)	4	(22.2)	0.58	1.54	0.47–5.03	33	(28.7)	4	(36.4)	0.73	0.70	0.19–2.57
Second line treatment, no. of cases (%)														
CR, no. of cases (%)	57	(69.5)	11	(73.3)	1.00	0.83	0.24–2.86	64	(71.9)	4	(50.0)	0.23	2.56	0.59–11.03
RR (CR+R), no. of cases (%)	79	(96.3)	13	(86.7)	0.17	4.05	0.62–26.62	85	(95.5)	7	(87.5)	0.36	3.04	0.30–30.98
Prednisolone therapy, no. of cases (%)	68	(63.0)	14	(77.8)	0.29	0.49	0.15–1.58	76	(66.1)	6	(54.5)	0.51	1.62	0.47–5.66
Response to prednisolone therapy														
CR, no. of cases (%)	37	(54.4)	8	(57.1)	1.00	0.90	0.28–2.86	42	(55.3)	3	(50.0)	1.00	1.24	0.23–6.52
RR (CR+R), no. of cases (%)	63	(92.6)	11	(78.6)	0.13	3.44	0.72–16.49	68	(89.5)	6	(100)	1.00		
Splenectomy, no. of cases (%)	17	(15.7)	2	(11.1)	1.00	1.50	0.32–7.10	18	(15.7)	1	(9.1)	1.00	1.86	0.22–15.40
Response to splenectomy														
CR, no. of cases (%)	10	(58.8)	1	(50.0)	1.00	1.43	0.08–26.90	11	(61.1)	0	(0)	0.42		
RR (CR+R), no. of cases (%)	13	(76.5)	1	(50.0)	0.47	3.25	0.16–64.61	14	(77.8)	0	(0)	0.26		
Eradication of *Helicobacter pylori*, no. of cases (%)	40	(37.0)	3	(16.7)	0.11	3.03	0.83–11.12	38	(33.0)	5	(45.5)	0.51	0.61	0.17–2.12
Response to eradication of *Helicobacter pylori*														
CR, no. of cases (%)	19	(47.5)	1	(33.3)	1.00	1.81	0.15–21.59	17	(44.7)	3	(60.0)	0.65	0.54	0.08–3.61
Severe ITP, no. of cases (%)	23	(21.3)	1	(5.6)	0.19	4.60	0.58–36.41	22	(19.1)	2	(18.2)	1.00	1.07	0.22–5.28
Refractory ITP, no. of cases (%)	9	(8.3)	1	(5.6)	1.00	1.55	0.18–12.99	9	(7.8)	1	(9.1)	1.00	0.85	0.10–7.40
Corticosteroid-dependent, no. of cases (%)	30	(28.8)	7	(41.2)	0.40	0.58	0.20–1.66	35	(30.4)	2	(18.2)	0.72	1.84	0.37–9.13

Second line treatment: Patients in need of second line, CR (complete response): platelet count of at least 100 × 109/L, R (response): platelet count between 30 and 100 × 109/L and at least double the baseline count, severe ITP: presence of bleeding symptoms at presentation sufficient to mandate treatment, or occurrence of new bleeding symptoms requiring additional therapeutic intervention with a different platelet-enhancing agent or an increased dose, refractory ITP: failure to achieve at least R or loss of R after splenectomy, loss of R: platelet count 30 × 109/L or a less than 2-fold increase in platelet count from baseline or the presence of bleeding, corticosteroid-dependence: the ongoing need for continuous corticosteroid administration or frequent courses of corticosteroids to maintain a platelet count at or above 30 × 109/L and/or to avoid bleeding.

*ITP* immune thrombocytopenia, *IFN* interferon, *IL* interleukin, *OR* odds ratio, *CI* confidence interval, *SD* standard deviation

**Table 6:** Treatment response of patients with chronic ITP with IL-4 and IL-4Rα

	IL4 CC		IL4 non-CC					IL4Rα QQ		IL4Rα non-QQ				
	*N*	(%)	*N*	(%)	*P* value	OR	95% CI	*N*	(%)	*N*	(%)	*P* value	OR	95% CI
All treatment, no. of cases (%)	14	(82.4)	83	(76.1)	0.76	1.39	0.37–5.22	71	(80.7)	26	(68.4)	0.10	2.08	0.87–4.97
Second line treatment, no. of cases (%)	9	(52.9)	28	(25.7)	0.04	3.25	1.15–9.25	29	(33.0)	8	(21.1)	0.21	1.84	0.75–4.52
Response to all treatments														
CR, no. of cases (%)	9	(64.3)	59	(71.1)	0.75	0.73	0.22–2.41	50	(70.4)	18	(69.2)	1.00	1.06	0.40–2.81
RR (CR+R), no. of cases (%)	13	(92.9)	79	(95.2)	0.55	0.66	0.68–6.36	68	(95.8)	24	(92.3)	0.61	1.89	0.30–12.00
Prednisolone therapy, no. of cases (%)	12	(70.6)	70	(64.2)	0.79	1.34	0.44–4.08	62	(70.5)	20	(52.6)	0.05	2.15	0.98–4.70
Response to prednisolone therapy														
CR, no. of cases (%)	5	(41.7)	40	(57.1)	0.36	0.54	0.16–1.85	34	(54.8)	11	(55.0)	1.00	0.99	0.36–2.74
RR (CR+R), no. of cases (%)	10	(83.3)	64	(91.4)	0.33	0.47	0.08–2.65	56	(90.3)	18	(90.0)	1.00	1.04	0.19–5.60
Splenectomy, no. of cases (%)	5	(29.4)	14	(12.8)	0.14	2.83	0.87–9.24	15	(17.0)	4	(10.5)	0.43	1.75	0.54–5.66
Response to splenectomy														
CR, no. of cases (%)	4	(80.0)	7	(50.0)	0.34	4.00	0.35–45.38	7	(46.7)	4	(100)	0.10		
RR (CR+R), no. of cases (%)	5	(100)	9	(64.3)	0.26			10	(66.7)	4	(100)	0.53		
Eradication of *Helicobacter pylori*, no. of cases (%)	7	(41.2)	36	(33.6)	0.59	1.38	0.49–3.93	33	(37.9)	10	(27.0)	0.24	1.65	0.71–3.84
Response to eradication of *Helicobacter pylori*														
CR, no. of cases (%)	4	(57.1)	16	(44.4)	0.69	1.67	0.33–8.55	15	(45.5)	5	(50.0)	1.00	0.83	0.20–3.44
Severe ITP, no. of cases (%)	5	(29.4)	19	(17.4)	0.32	1.97	0.62–6.26	19	(21.6)	5	(13.2)	0.33	1.82	0.62–5.29
Refractory ITP, no. of cases (%)	0	(0)	10	(9.2)	0.36			9	(10.2)	1	(2.6)	0.28	4.22	0.52–34.51
Corticosteroid-dependent, no. of cases (%)	5	(33.3)	32	(30.2)	0.77	1.16	0.37–3.66	28	(33.3)	9	(24.3)	0.39	1.56	0.65–3.74

Second line treatment: Patients in need of second line, CR (complete response): platelet count of at least 100 × 109/L, R (response): platelet count between 30 and 100 × 109/L and at least double the baseline count, severe ITP: presence of bleeding symptoms at presentation sufficient to mandate treatment, or occurrence of new bleeding symptoms requiring additional therapeutic intervention with a different platelet-enhancing agent or an increased dose, refractory ITP: failure to achieve at least R or loss of R after splenectomy, loss of R: platelet count 30 × 109/L or a less than 2-fold increase in platelet count from baseline or the presence of bleeding, corticosteroid-dependence: the ongoing need for continuous corticosteroid administration or frequent courses of corticosteroids to maintain a platelet count at or above 30 × 109/L and/or to avoid bleeding.

*ITP* immune thrombocytopenia, *IFN* interferon, *IL* interleukin, *OR* odds ratio, *CI* confidence interval, *SD* standard deviation

### Th1/Th2 ratio in patients with cITP and healthy controls

**Fig. 1:**
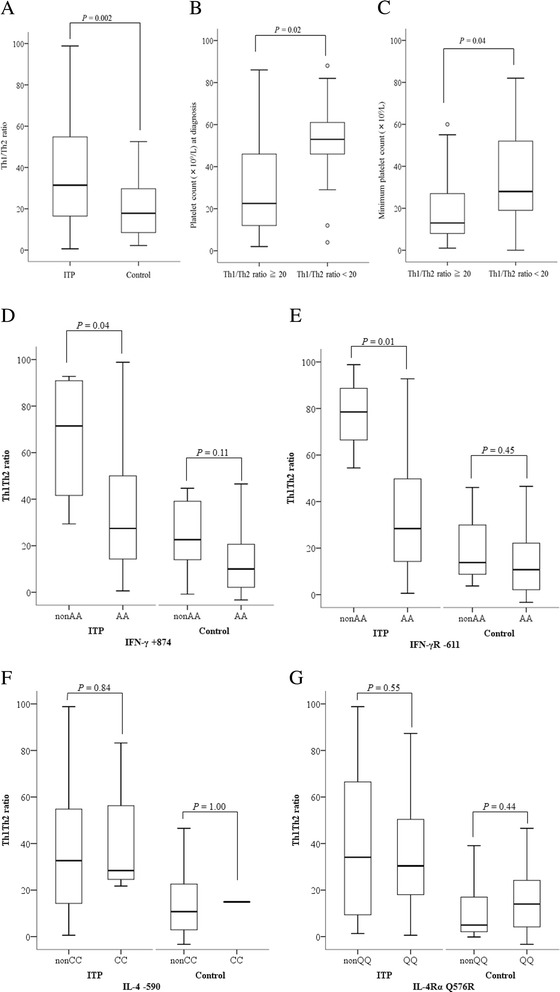
Th1/Th2 ratio of patients with chronic ITP and healthy controls. **a** Th1/Th2 ratio of patients with chronic ITP was significantly higher than healthy controls (median, 31.4 vs. 17.8, P = 0.002). **b** Platelet count (×10^9^⁄L) at diagnosis of chronic ITP with Th1/Th2 ratio ≧ 20 was significantly lower than that with Th1/Th2 ratio < 20 (median, 22.5 vs. 53.0, *P* = 0.02). **c** Minimum platelet count (×10^9^⁄L) of chronic ITP with Th1/Th2 ratio ≧ 20 was significantly lower than that with Th1/Th2 ratio < 20 (median, 13.0 vs. 28.0, P = 0.04). **d** Th1/Th2 ratio of patients with the IFN-γ +874 non-AA genotype was significantly higher than that of the AA genotype (median, 71.5 vs. 27.5, P = 0.04). Th1/Th2 ratio of controls was similar in IFN-γ +874 polymorphism. **e** Th1/Th2 ratio of patients with the IFN-γR −611 non-AA genotype was significantly higher than that of the AA genotype (median, 78.5 vs. 28.4, *P* = 0.01). Th1/Th2 ratio of controls was similar in IFN-γR −611 polymorphism. **f** Th1/Th2 ratio of both patients and controls with the IL-4–590 non-CC genotype was not significantly different from that of the CC genotype. **g** Th1/Th2 ratio of both patients and controls with the IL-4Rα Q576R non-QQ genotype was not significantly different from that of the QQ genotype

The median Th1/Th2 ratio in patients with cITP was significantly higher than that of healthy controls (31.4, range 0.6–98.8 vs. 17.8, range, 2.2–52.5 respectively; *P* = 0.002) (Fig. 1a). As the median Th1/Th2 ratio was approximately 20 in the control group, we divided cITP patients into 2 groups; high Th1/Th2 (Th1/Th2 ratio ≥20) and low Th1/Th2 (Th1/Th2 ratio <20). The high Th1/Th2 group had a significantly lower platelet count at diagnosis than the low Th1/Th2 group (22.5 × 10^9^⁄L, range 4.0–88.0 × 10^9^⁄L vs. 53.0 × 10^9^⁄L, range 2.0–86.0 × 10^9^⁄L, respectively; *P* = 0.02) (Fig. 1b). The minimum platelet count during the clinical course in the high Th1/Th2 group was significantly lower than in the low Th1/Th2 group (median 13.0 × 10^9^⁄L vs. 28.0 × 10^9^⁄L respectively, *P* = 0.04) (Fig. 1c). These data suggest a role for the Th1/Th2 ratio in the pathogenesis of cITP.

### Th1/Th2 ratio of both patients and healthy controls with IFN-γ, IFN-γR, IL-4, and IL-4Rα polymorphisms

Patients with the *IFN-γ* +874 non-AA genotype (high expression type) had a significantly higher Th1/Th2 ratio compared to those with the *IFN-γ* +874 AA genotype (low expression type) (71.5 [range 29.4–92.8] vs. 27.5 [range 0.6–98.8] respectively; *P* = 0.04) (Fig. 1d). Furthermore, patients with the *IFN-γR −*611 non-AA genotype (high-function type) had a significantly higher Th1/Th2 ratio compared to those with the *IFN-γR −*611 AA genotype (low function type) (medians, 78.5 vs. 28.4 respectively, *P* = 0.01) (Fig. 1e). However, there was no significant association between the Th1/Th2 ratio and *IL-4/IL-4Rα* polymorphisms (Fig. 1f, g). In contrast to cITP, there was no significant association between the Th1/Th2 ratio and all these four polymorphisms in the control group (Fig. 1d, e, f, g). These data confirm that Th1 polarization due to *IFN-γ* and *IFN-γR* polymorphisms is associated with the Th1/Th2 ratio in cITP.

## Discussion

Although the immune mechanism that initiates cITP has not been identified, several processes of immune dysregulation have been reported [22]. It is well known that autoantibodies to GpIb/IX and/or GPIIb/IIIa induce the destruction of platelets in peripheral blood, as well as the production of platelets in bone marrow. T cell abnormalities have also been reported in cITP, including a high Th1/Th2 ratio, a high cytotoxic T cell type 1/cytotoxic T cell type 2 lymphocyte ratio, high Th17 cell levels, and decreased Treg cells. Furthermore, genetic factors such as polymorphisms of the cytokine genes *FcgR* and *HLA* were reported to contribute to the pathogenesis of cITP. Thus, cITP is considered to be a consequence of complex immune dysregulation events in conjunction with the presence of genetic risk factors.

Recent studies using flow cytometry and real-time PCR have revealed a clear Th1-polarized cytokine profile both at the protein and mRNA levels in cITP [2, 3, 5, 10]. However, it is unclear whether fluxes in the Th1/Th2 ratio that lead to cITP pathogenesis involve genetic factors.

Numerous studies have shown that patients with autoimmune disease have polarized Th1 or Th2 responses [23]. IFN-γ is one of the main cytokines used to distinguish Th1 from other CD4+ subsets. IFN-γ, which is secreted mainly by Th1 and natural killer cells, promotes inflammation, responses to intracellular pathogens, and switching to the IgG2a, IgG2b, and IgG3 subclasses [24, 25]. IFN-γ exerts its biological effect by binding to the IFN-γ receptor (IFNGR), which consists of two chains: a receptor α chain (IFNGR1) and a receptor β chain (IFNGR2). Dysregulated IFN-γ production has been reported in many autoimmune diseases, including Hashimoto’s disease, type I diabetes, and multiple sclerosis. Panitsas et al. showed that serum levels and leukocyte gene expression of IFN-γ are markedly elevated in patients with cITP [3]. A high Th1/Th2 ratio showing a Th1-polarized response was also reported in cITP patients; this was reversed by treatment with dexamethasone [26, 27]. Thus, Th1 polarization may comprise a pivotal event in the pathogenesis of cITP [10].

Furthermore, recent studies have shown the association between *IFN-γ* +874 T/A polymorphism and various diseases such as cancer and autoimmune disorders. *IFN-γ* +874 T/A polymorphism has been reported to affect the production of IFN-γ; the TT genotype has been linked to higher production of IFN-γ compared to the A/A genotype [15, 28]. Although there are few studies on *IFN-γ* polymorphisms in cITP patients, they produced inconsistent findings. Pehlivan et al. [29] reported that cITP patients had a significantly higher frequency of the *IFN-γ* +874 TT genotype (high expression type) compared to healthy controls, while Chen et al. reported no significant association between *IFN-γ* +874 T/A polymorphism and infant ITP in Chinese patients [30]. We also found no significant association between *IFN-γ* +874 T/A polymorphism and Japanese patients with cITP. The frequency of *IFN-γ* +874 T/A polymorphism has been reported to differ by race [19, 20], which may explain the inconsistencies in various reports; in our control group, the rate was similar to that previously reported in healthy Asian control subjects [20]. Although Chen et al’s results were in accordance with our findings, they did not show the clinical characteristics of cITP according to *IFN-γ* +874 T/A polymorphism.

In contrast to cITP susceptibility, our data showed that cITP patients with *IFN-γ* +874 non-AA genotype (high expression type) had a lower minimum platelet count than those with the AA genotype. Panitsas et al. showed that lower peripheral platelet counts correlated with higher *IFN-γ* mRNA levels [3]. Thus, the patients with *IFN-γ* +874 non-AA genotype may be susceptible to severe cITP. Further studies may be needed to confirm the involvement of the *IFN-γ* gene in the pathogenesis of cITP.

IL-4 is the Th2 cytokine that is pivotal for the pathogenesis of many autoimmune diseases; it induces the differentiation of Th0 cells to Th2 cells [31]. Th2 cells subsequently produce additional IL-4 in a positive feedback mechanism upon activation by IL-4 [31]. There are some *IL-4* polymorphisms which affect the expression level of IL-4, including *IL-4* VNTR intron 3 and *IL-4*-590C/T. Rosenwasser et al. [32] analysed the association between IL-4 production and the *IL-4* -590C/T polymorphism, and reported the TT genotype was linked to higher IL-4 levels compared to the C/C genotype. Several *IL-4* -590C/T polymorphism studies were reported in various autoimmune diseases, including asthma, rheumatoid arthritis, and multiple sclerosis [33–35]. We found no association between *IL-4* -590C/T polymorphism and susceptibility to cITP. A similar finding has been reported by Foster et al. [36]. In contrast to *IL-4* -590C/T polymorphism, they produced inconsistent findings of the association between *IL-4* VNTR intron 3 and ITP susceptibility. Makhlouf et al. [37] reported that Egyptian cITP patients had a significantly association with *IL-4* VNTR, however Chen et al. reported showed no significant association between *IL-4* VNTR intron 3 and Chinese ITP patients [30, 37].

cITP patients with the *IL-4* -590 CC genotype (low expression type) had a higher number of treatment regimens than those with the non-CC genotype. Thus, the *IL-4* -590 CC genotype, which predominantly induced Th1, appears to contribute to poor response to treatment.

IL-4 exerts its biological effects via signalling through its receptor, IL-4R. There are two types of IL-4 receptor complexes; the type I receptor consisting of IL-4Rα and the common gamma chain, and the type II receptor consisting of IL-4Rα and IL-13Rα [38]. *IL-4Rα* Q576R, which can affect the binding of IL-4 and phosphorylation of intracellular substrates including signal transducer and activator of transcription 6 (STAT6), has been linked to many autoimmune disorders such as asthma, atopy, and allergy [39]. Our study demonstrated that cITP patients had significantly higher frequencies of the *IL-4Rα* 576 non-QQ genotype. To our knowledge, ours is the first report demonstrating that *IL-4Rα* Q576R influences susceptibility to cITP. Recently Massoud et al. have shown that *IL-4Rα* Q576R promotes conversion of induced Treg cells toward a Th17 cell fate using Asthma mouse model [40]. Many investigators have shown that ITP patients have higher Th17/Treg compared to normal controls [4]. Th17 polarization by *IL-4Rα* Q576R polymorphism may affect the susceptibility to cITP in our study.

In this study, cITP patients had a higher Th1/Th2 ratio compared to healthy subjects. Among cITP patients, those with a higher Th1/Th2 ratio had a significantly lower platelet count than those with a lower ratio; these results are consistent with previous studies [2, 3]. Our study showed that the patients with *IFN-γ* non-AA and *IFN-γR* non-AA genotypes, which are related to higher IFN-γ expression and higher IFN-γR function, respectively, had a significantly higher Th1/Th2 ratio (*P* < 0.05). Thus, such genotypes of the IFN-γ pathway components might contribute to a higher Th1/Th2 ratio in cITP patients, and may thus increase the severity of cITP. We also explored the association between these four polymorphisms and Th1/Th2 ratios in healthy control subjects; however, we found no significant association between these polymorphisms and Th1/Th2 ratios.

## Conclusion

In conclusion, our study revealed that the *IL-4Rα* polymorphism is associated with susceptibility to cITP. Moreover, the *IFN-γ* +874 non-AA genotype is associated with more severe thrombocytopenia and a higher Th1/Th2 ratio in cITP, indicating that the cytokine polymorphisms affecting Th1/Th2 increase the susceptibility to, and severity of, chronic ITP.

## Abbreviations

CI: Confidence interval
cITP: Chronic immune thrombocytopenia
IFN: Interferon
IFNGR: Interferon-γ receptor
IL: Interleukin
OR: Odds ratio
SD: Standard deviation
STAT6: Signal transducer and activator of transcription 6
Th1: T-helper cell type 1
Th2: T-helper cell type 2
Treg: Regulatory T
